# The Tenderness of Christ

**Published:** 1889-09-21

**Authors:** 


### Words of Consolation.

### THE TENDERNESS OF CHRIST.

In the numerous prophecies in the Book of Isaiah which speak of our Lord Jesus Christ, we are struck by the tender and compassionate character of Him who was coming in the fulness of time to be our Saviour. He is spoken of in one place as a "strength to the poor, a strength to the needy in his distress, a refuge from the storm, a shadow from the heat." In another, the words which Christ afterwards applies to Himself in the synagogue at Capernaum runs thus: "The Spirit of the Lord is upon me because He hath anointed me to preach good tidings to the meek; He hath sent me to bind up the broken-hearted, to proclaim liberty to the captives, and the opening of the prison to them that are bound." Here all are comprehended—the weak, the poor, the sick, the sad, the lonely. Everyone may come to Him as to a pool of Bethesda, whose waters are always troubled and ready to heal. Nay, not only ready, but overflowing to any who show the slightest wish to avail themselves of the cure. Again, we read, "A bruised reed shall He not break, and the smoking flax shall He not quench." He strengthens the weak hands, and confirms the feeble knees, and says to them that are of a fearful heart, Be strong, fear not, your God will come and save you. What blessed promises to those who should be living when our dear Lord was upon earth! Were they accomplished? Undoubtedly so. He who giveth all things to all men liberally did not then stint His hand He drove none to despair, He gave neither frown nor harsh reproof, scarcely ever upbraided His disciples, from whose waywardness He had so much to bear. When they would have called down fire from heaven upon the Samaritan cities which would not receive Him, it was only with a softened melancholy tenderness that He said, "Ye know not what manner of spirit ye are of," and the boastful, ambitious souls were abashed by, "What was it that ye disputed among yourselves by the way?" The wondrous look of mingled love, sorrow, and disappointment which our Lord cast on Peter after his denial, and the gentle words, "Simon, son of Jonas, lovest thou Me?" were the only signs of the bitter grief such ingratitude must have caused. How keenly it would have tried us to see those who professed to love us able to sleep on through our anguish, leaving us to suffer unhelped, unpitied, and alone. The tender Saviour excuses all. It was not indifference which closed the disciples' eyes—they were "sleeping for sorrow." Want of sympathy, there was none. "The spirit indeed is willing, but the flesh is weak." He was forward to make excuses for their cowardly flight and unfeeling conduct at His betrayal, for through His angel He bade the women at the sepulchre tell "His disciples *and Peter* that He had risen," thus relieving their minds from the guilt and misery which must have weighed them down.

Nor was He less tender and compassionate to those who were not His followers. The fainting multitudes in the wilderness were fed; the woman at the well of Samaria and the penitent who washed His feet with her tears and wiped them with the hairs of her head were pardoned and encouraged; and the adulteress was told to go and sin no more. Wherever He went the same loving compassion went too. He grieved over the hardness of men's hearts which prevented him working miracles at Nazareth, and wept over Jerusalem because her people knew not the day of their visitation.

In heaven He now stands in glory making intercession for us as the High Priest who can be touched with the feeling of our infirmities.

And what our Blessed Lord did then on earth He can and will do now; He is the same yesterday, to-day, and for ever. Are we needy or in distress, longing for a refuge from the storm? Are we broken-hearted or feeling the awful burden of sin? Carry thy sorrows to Jesus, and fear not, weak one, though thy faith be small; thy safety depends not on thine own weak efforts. The Eternal God is thy Refuge, and underneath are the Everlasting Arms. He will sanctify and preserve thee for ever, so that neither life nor death, health nor sickness, prosperity nor adversity may ever separate thee from the love of God, which is in Christ Jesus thy Lord.

